# Combined Aplasia of Sphenoid, Frontal, and Maxillary Sinuses With Hypoplasia of The Ethmoid Sinus

**DOI:** 10.5812/ircmj.2627

**Published:** 2013-01-05

**Authors:** Tolga Kandogan, Abdullah Dalgic, Hulya Mollamehmetoglu, Ozgur Esen

**Affiliations:** 1 Department of Otolaryngology, Izmir Teaching Hospital, Izmir, Turkey; 2 Department of Radiology, Izmir Teaching Hospital, Izmir, Turkey

**Keywords:** Aplasia, Hypoplasia, Paranasal sinus, Sphenoid sinus

**Figure 1:**
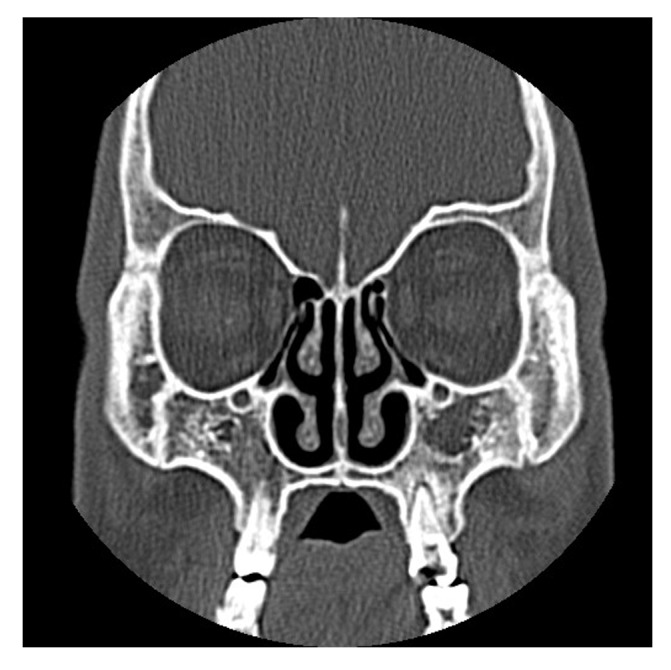
Paranasal Sinus CT, Coronal Section, Indicating Aplasia of the Maxillary Sinus and Minimal Pneumatization in the Ethmoid Cells.

Dear Editor,

Paranasal sinuses are prone to a great diversity of anomalies. Aplasia of the frontal sinus is a relatively common phenomenon and may be accompanied with hypoplasia of the maxillary sinus, but combined aplasia or underdevelopment of the paranasal sinuses is unusual (1, 2).These developmental pathologies may be misdiagnosed as sinusitis or neoplasm (2). Computerized Tomography (CT) is an excellent imaging technique that is used to map sinus anatomy (3). Here, we present a 23-years old male patient who has combined aplasia of sphenoid, frontal, and maxillary sinuses accompanied by ethnocide sinus hypoplasia, which is the second reported case to our knowledge. Twenty three years old male patient presented with complaints of headache and nasal obstruction to the Otorhinolaryngology outpatient clinic. Axial and Coronal CT scans (5mm slice thickness) delineated lack of pneumatization in the frontal, sphenoid and maxillary sinuses and minimal pneumatization in the ethmoid cells eg. Total aplasia of the frontal, sphenoid and maxillary sinuses and hypoplasia of the ethmoid sinuses. (Figure 1) No other craniofacial anomaly was observed. He had no previous history of facial trauma, irradiation or systemic diseases affecting the skeletal system such as Paget’s disease, osteopetrosis, or fibrous dysplasia. No other abnormality was found on clinical and laboratory examinations, including cystic fibrosis. The paranasal sinuses begin their development as an evagination of the mucosa from the nasal cavities during the third and fourth fetal months. They undergo major expansion after birth, along with the development of the facial cranium and teeth (1) The underdevelopment or aplasia of the paranasal sinuses is a rare phenomenon that refers mainly to the frontal (12%) and secondarily to the maxillary sinuses (5% and 6%). Agenesis of the sphenoid sinuses is extremely rare (4). The agenesis of the paranasal sinuses occurs more frequently in syndromes of craniosynostosis, osteodysplasia (Melnick-Needles), as well as in cases of Down’s syndrome (hypoplasia of the frontal sinus) (3). Developmental anomalies of paranasal sinuses in cystic fibrosis patients is significantly high compared to normal population (3). The developmental pathologic abnormalities may be misdiagnosed as sinusitis or neoplasm (4). The maxillary sinus is the first of the paranasal sinuses to develop in the human fetus and is present at birth. Development of the maxillary sinus may become arrested in children with recurrent chronic rhino sinusitis (4). For many authors there is a connection between reduced nasal ventilation and abnormal maxillary growth, but Diner et al observed normal development of maxillary sinus in 11 patients with unilateral choanal atresia. Chronic obstruction due to narrow infundibular passage and absence of natural osmium leads to thick effusion in hypo plastic sinuses (2, 4)Maxillary sinus hypoplasia may be misdiagnosed as chronic sinusitis. The sphenoid sinus develops embryo logically from the skull base. By the age of 12, the sphenoid pneumatization reaches its final form to a size equivalent to that of an adult. Sphenoid sinuses vary in size and shape. The average measurements of the sphenoid sinus are as follows: vertical height, 2 cm; transverse breadth, 1.8 cm; and anterosuperior depth, 2.1 cm. Hypoplasia of the sphenoid sinuses is shown frequently in patients with cystic fibrosis. However, agenesis of the sphenoid sinus is highly uncommon (4). Based on their anatomical studies, Wertheim and Grunwald reported in the early 1900s that agenesis of the sphenoid sinuses can indeed occur in 1–1.5% of cases. The diagnosis of sphenoid sinus hypoplasia is potentially important in patients in whom trans-spheroidal hypophysectomy is contemplated. In conclusion this patient seems to be the second case to have combined aplasias of the sphenoid, frontal, and maxillary sinuses with hypo plastic ethmoid cells without any systemic or skeletal disease. This extremely rare anomaly should also be kept in mind to prevent complications during endoscopic sinus surgery.
